# Temperature‐Dependent Chain Structures during Solution‐Grown Crystallization via Atomic Force Microscopy

**DOI:** 10.1002/marc.202500842

**Published:** 2026-01-26

**Authors:** Dingrui Wang, Xiaobin Liang, Ken Nakajima

**Affiliations:** ^1^ Department of Chemical Science and Engineering School of Materials and Chemical Technology Institute of Science Tokyo Ookayama 2‐12‐1, Meguro‐ku Tokyo 152‐8552 Japan

**Keywords:** chain structure, crystallization mechanism, single polymer chain, single‐molecule force spectroscopy

## Abstract

Semi‐crystalline polymers are widely utilized due to their favorable cost, and their crystallization behavior is significantly influenced by crystallization temperature. In this study, we employed AFM‐based SMFS (single‐molecule force spectroscopy) to investigate single‐chain folding structures in Polyethylene Oxide (PEO) at various crystallization temperatures (*T_c_*) to elucidate the temperature effect. We utilized force‐volume (FV) mode for SMFS, which enables the measurement of single‐chain information while simultaneously obtaining information about the surface morphology of the crystal. By comparing the morphological changes before and after SMFS, we can locate the position of the stretched single chain and observe the morphological changes after the single chain is stretched. Combined with the force‐extension curves of a single polymer chain, we can infer information about the 3D structure of the molecular chains and the interchain structure in the crystal. Morphological and molecular‐level structural data show that intermediate structures are more likely to form at low temperatures, while adjacent reentry structures form at high temperatures. Interestingly, temperature does not affect the degree of chain folding, i.e., the number of <n> adjacent reentry folds. However, the aggregation structures formed by chain folding differ due to changes in surface free energy. This provides a new method for studying the structural changes of a single chain during polymer crystallization.

## Graphical abstract

AFM‐based single‐molecule force spectroscopy was employed to investigate temperature‐dependent single‐chain folding structures of semi‐crystalline PEO. Force–volume measurements enabled correlation of force–extension behavior with local crystal morphology. Results reveal temperature‐dependent aggregation structures, with intermediate structures favored at low temperatures and adjacent reentry structures at high temperatures, while the degree of chain folding remains unchanged.

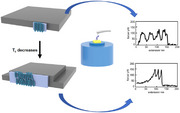

## 1 Introduction

Semi‐crystalline polymers, including polyethylene, polypropylene, and poly(ethylene oxide), are widely used in various applications due to their favorable cost [1]. The properties of semi‐crystalline polymers are primarily influenced by the chain structures formed during different processing conditions, with temperature being an important factor [2, 3]. Therefore, understanding chain structure is essential for investigating the crystallization process and developing semi‐crystalline polymers with enhanced properties. Various models have been proposed by researchers to explain the changes of trajectory of polymer chains during the crystallization process and the effects of temperature. For example, the traditional Lauritzen‐Hoffman (LH) model, based on the secondary nucleation mechanism, predicts the kinetics of a process in which stems with lengths equal to the lamella thickness are deposited on the growth front while chain folding occurs simultaneously. This model explains various experimental results of polymer crystallization. The microscopic mechanism of the three different crystallization behaviors is revealed based on the temperature‐dependent competition between chain nucleation and lateral growth processes, referred to as the three regime theory [4, 5, 6]. To further extend the LH model at the molecular level, Sadler and Gilmer discussed kinetic barriers caused by molecular continuity, making the model applicable to various molecular structures [7]. Furthermore, Keller et al. proposed that crystals can appear and grow in a mesophase during crystal growth [8]. Similarly, Strobl found that polymer crystallization generally follows a route that includes a passage through a mesomorphic phase [9, 10, 11]. However, as researchers continued to study and experiment with the crystallization mechanism, it was found that the classical LH theory fails over a wide range of temperatures. The LH theory assumes that crystalline thickness would suddenly increase at very large supercoolings. But the crystalline thickness decreases and eventually stabilizes at a constant lowest value at large supercoolings [12]. Additionally, Point and Janimak pointed out that the values of substrate length and kinetic length in the three‐regime theory were unreasonable [13]. Additionally, Gao also provided three crystallization states with decreasing temperature and examined the influence of chain length on the crystallization process, yielding results contrary to the traditional LH model [14]. In summary, polymers exhibit complex crystallization behavior at different crystallization temperatures, making it necessary to characterize the crystallization structure at the microscopic scale to gain further insight into the crystallization kinetics of polymer materials.

For a long time, researchers have tried to uncover the microscopic mechanism of the crystallization process through various experimental methods. For instance, fourier‐transform infrared spectroscopy (FT‐IR), wide‐angle X‐ray scattering (WAXS), and small‐angle X‐ray scattering (SAXS) [15, 16, 17, 18, 19, 20] have observed that chains fold to form pre‐ordered structures in the early stages of crystallization, indicating the aggregation model that polymer chains may self‐fold first and aggregate subsequently to form regular lamellae [21, 22]. Recently, with advanced characterization techniques, it has become possible to study crystallization behavior and clarify crystallization mechanisms at the single polymer chain level. For example, solid‐state nuclear magnetic resonance (SS‐NMR) has studied various chain structures within crystals by comparing DQ signal intensities from different chain folding models, and first found chain folding structures in both melt‐ and solution‐grown with varied temperatures [23, 24, 25, 26]. It revealed the structure and properties of an intermediate part between the crystalline lamellae and the isotropic amorphous fraction (IAF) during melt recrystallization of PVDF [27]. The Kumaki group utilized in situ atomic force microscopy (AFM) to observe the crystallization process of a folded‐chain crystal of it‐PMMA under high humidity at the molecular level [28]. In particular, Zhang et al. used single‐molecule force spectroscopy (SMFS) to pull a single polymer chain from a single crystal and simultaneously measure the intermolecular and intramolecular interactions received during the unfolding of the single polymer chain [29]. Analysis of the force‐extension curves obtained by SMFS during the extension of the single chain provides a deeper understanding of the folded structure of the single chain and the mechanism of its folding behavior [30, 31, 32, 33]. SMFS has enabled the characterization of individual polymer chains in single crystals, representing a significant development in the study of the crystallization behavior of polymers. However, most existing research focuses on the unfolding process of folded chains in single crystals, while little attention has been paid to the spatial conformation of molecular chains in the multilayer crystals.

**FIGURE 1:**
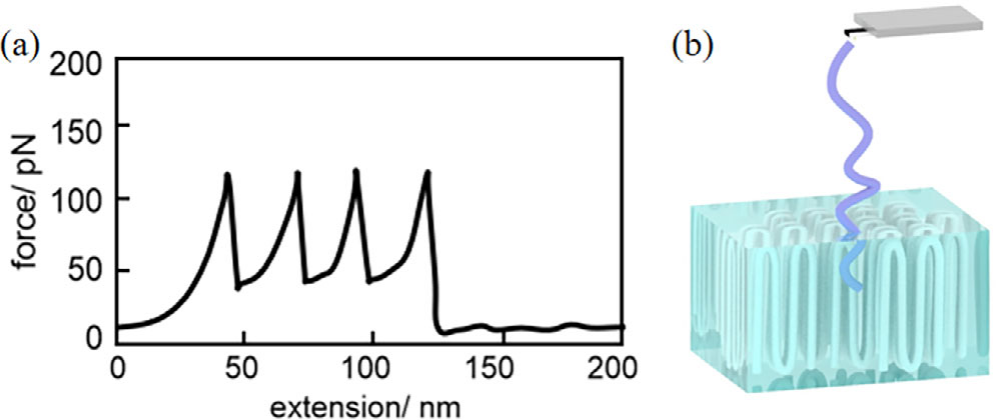
(a) Force‐extension curves and (b) its corresponding chain structure in the crystalline region.

Therefore, in this study, SMFS experiments were performed in force‐volume mode to measure the force‐extension curve of a single polymer chain being stretched while tracking changes in the crystallographic surface [34, 35]. By comparing the changes in the microtopography of the crystallographic surface before and after the polymer chain was pulled out, we obtained the spatial conformation of a single polymer chain in the crystal and inferred further microscopic information about the stacking structure of the polymer folding chain in the crystal, which supports that the adjacent reentry structures are independent of *T_c._* [36]. This is crucial for understanding the microscopic mechanism of polymer crystallization. Here, we conducted SMFS experiments on single crystals of polyethylene oxide (PEO) formed at different crystallization temperatures (*T_c_*), focusing on changes in their chain structures. Figure 1a illustrates the force‐extension curve of a typical single chain stretched out of the crystal, where multiple peaks correspond to adjacent reentry folded chains, representing the stretching and unfolding of individual chains. The distance between each peak is approximately twice the thickness of the single crystal, representing the unfolding of an adjacent reentry chain. We can, therefore, speculate that this stretched single chain is a folded chain structure in crystallization, as shown in Figure 1b. Furthermore, the force‐extension curve combined with morphological information can infer the spatial conformation of single molecular chains in polymer crystallization. We used this method to study the structure of polymer chains and their temperature‐dependent behavior during crystallization.

## 2 Results and Discussion

### 2.1 Effect of Temperature on Crystal Morphology

**FIGURE 2:**
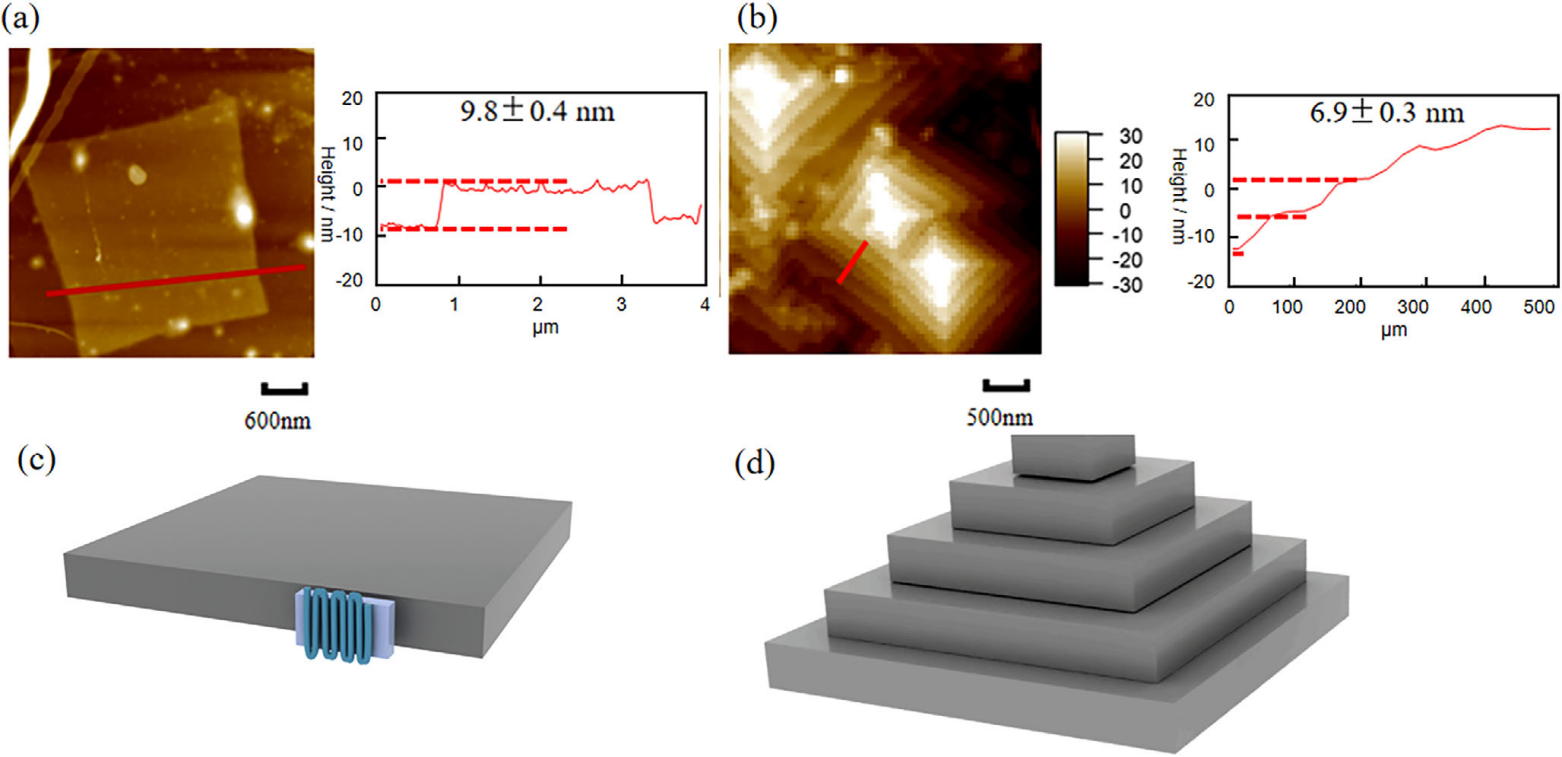
Height images measured by AFM tapping mode were obtained from crystals formed by isothermal crystallization for 10 h at *T_c _= *(a) 35°C and (b) 10°C in air, with the thickness of the monolayer estimated from the height change. The chains in the monolayer crystal adopt (c) a regular adjacent reentry model, while (d) in the multilayer crystal, the structure is still under debate.

Figure 2 displays the morphology of PEO single crystals formed at *T_c _= *35°C and 10°C, measured by AFM in air. The single crystals formed at 35°C exhibit a uniform thickness and a regular single‐layer square morphology, with a thickness of approximately 9.8 ± 0.4 nm, as shown in Figure 2a. As the *T_c_* decreases to 10°C, the single crystals remain square, but the thickness decreases to about 6.9 ± 0.3 nm, forming multilayer crystals, as shown in Figure 2b. Additionally, there is a significant difference in crystal size at different crystallization temperatures. Compared to high‐temperature crystallization, the number of lamellar crystals formed at 10°C is significantly higher, and the 2D size of each layer decreases to 1–2 µm, which is much smaller than the size of the monolayer at 35°C. This temperature dependence of crystallization morphology has been observed in many studies; the thickness of the lamellar crystals increases, and the 2D size increases as the degree of supercooling decreases due to an increase in crystallization temperature [12, 37]. In the classical LH theory, this difference in crystalline morphology is the result of competition between nucleation and crystalline growth. A limited number of nuclei are formed at low supercooling, and only a single crystal domain can be generated. Conversely, nucleation occurs frequently at large degrees of supercooling, and many localized nuclei are continuously formed at different sites, generating multilayered crystals [38].

The microstructure of single molecular chains in monolayer crystals is usually considered to follow the adjacent reentry model (Figure 2c), and its corresponding force‐extension curves are similar to the curve in Figure 1a. However, in multilayer crystals formed by low‐temperature crystallization, the microstructure becomes more complex and remains under debate (Figure 2d). Two main possible models exist for this multilayer crystalline structure. The first model is the adjacent reentry model, in which multiple nuclei grow in the same plane, and the crystal chains are folded in the 2D direction to form a monolayer structure of uniform thickness, which is the same as that of the monocrystal formed at 35°C. The monolayers are then stacked on top of each other to form multilayer crystals. The second model is the intermediate model, in which the nucleus grows in a 3D direction, and single molecular chains are interspersed between the layers during the folding process, resulting in complex multilayers with interlocking crystalline layers stacked layer by layer. However, existing molecular dynamics simulations and traditional characterization methods can only reveal morphological differences and cannot directly resolve the 3D chain‐level structure of polymer chains. As the chain folding mode within these multilayered crystals remains controversial, in the next section, we investigate the 3D microstructures of polymer chains in depth using SMFS.

### 2.2 Chain‐folding Analysis by SMFS for High‐Temperature Crystallization

**FIGURE 3:**
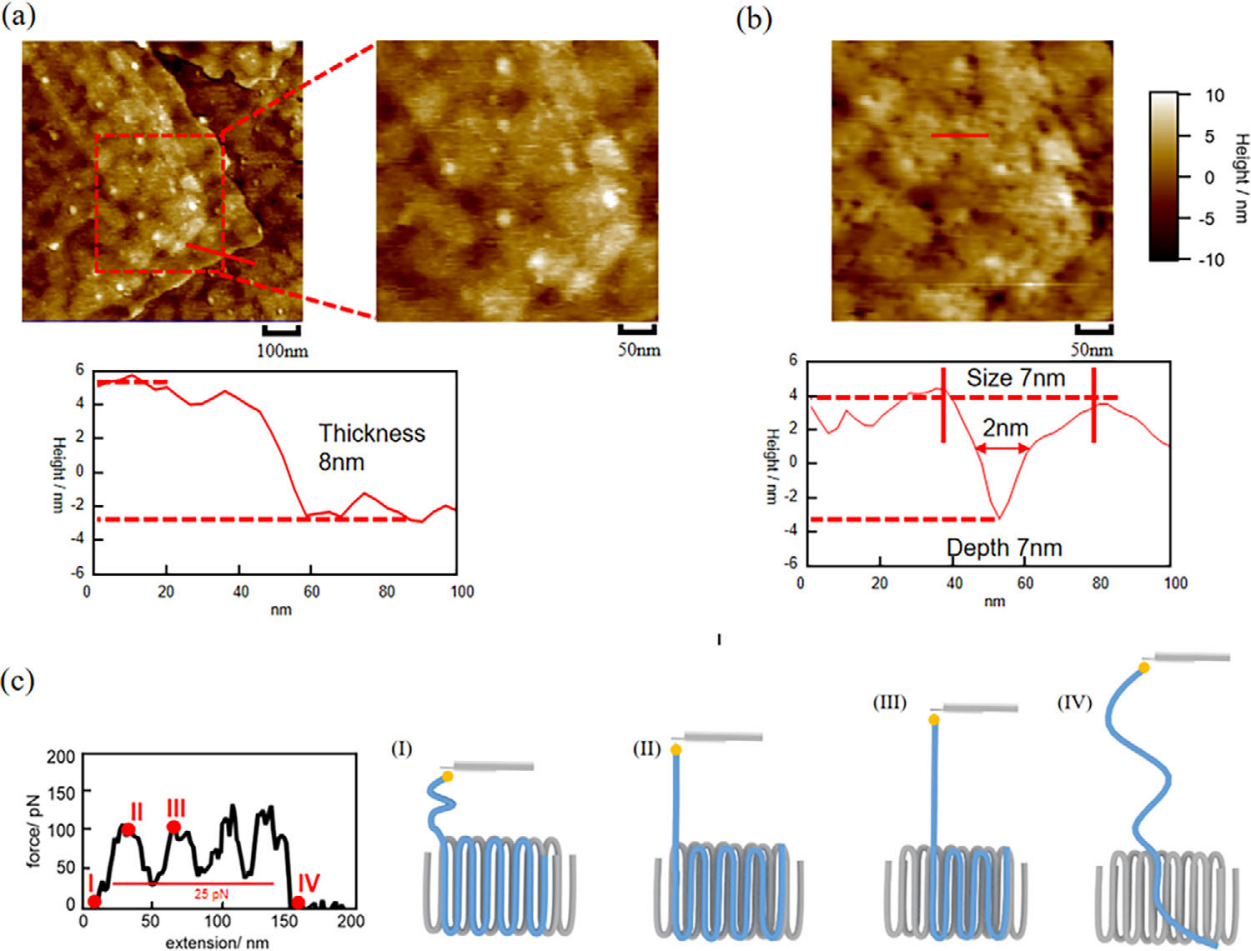
The morphology of the crystal formed at *T_c _= *35°C (a) before SMFS and (b) after SMFS in amyl acetate (liquid), along with their corresponding thickness information. (c) Force‐extension curve of a single chain in amyl acetate obtained in a single crystal formed at *T_c _=* 35°C, and four steps of a single chain stretched by a gold‐coated cantilever in this sample.

SMFS has garnered significant attention due to its ability to stretch individual polymer chains from single crystals and analyze the conformational structure and interchain interactions of folded chains through force‐extension curves. However, a major limitation of current SMFS experimental methods is the difficulty in precisely determining the spatial location of the polymer chain being stretched. This restricts our understanding of the 3D conformation of folded chains within crystalline structures. To address this issue, we first used tapping mode in liquid to obtain high‐resolution morphological images of the selected single‐crystal regions. Subsequently, we switched to FV mode to conduct quick SMFS experiments in the selected regions, thereby obtaining corresponding force‐extension curves. Finally, we switched back to tapping mode to capture the same region again, allowing a direct comparison before and after SMFS experiments to extract more detailed structural information. Figure 3a presents the morphology of a single crystal formed at *T_c _= *35°C before SMFS experiments, obtained via tapping mode in liquid. The image reveals a flat and well‐defined crystal surface, exhibiting a shape and thickness similar to that of the single crystal measured in air (Figure 2a). Interestingly, several nanometer‐scale granular structures (white spots) were observed on the surface. We speculate that these structures may be caused by crystallization defects because the molecular chains fail to form a completely regular folding chain when folding with double thiol ends. Notably, after multiple SMFS experiments in this region, the white granular structures almost completely disappeared, while numerous depressed structures (black spots) emerged on the surface. Depth analysis of these black spots indicates an average depth of approximately 7 nm, which corresponds well with the thickness of folded chains. This suggests that these black spots are voids left behind after individual polymer chains were extracted from the single crystal. Furthermore, the disappearance of the granular structures implies that they originated from irregularly folded regions at polymer chain termini (thiol‐terminal groups, which may lead to larger unfolded regions). Since SMFS can effectively capture the thiol groups at the free polymer ends, these molecular chains can be pulled out more easily. The force‐distance curves obtained from SMFS experiments in this region reveal that 34 polymer chains were stretched, some in the defect regions and some in the defect‐free regions. This number aligns with the number of black spots observed in Figure 3b (approximately 30). This correspondence suggests that each black spot represents a single stretched polymer chain. It is worth noting that the lateral size of the depressed structures is significantly larger than the theoretical size of a single folded chain, a discrepancy is likely due to the probe radius, which prevents precise measurement of the actual dimensions of individual chains. When a single polymer chain is stretched from the crystalline region, only a small void is left at its original location, while the surrounding structure remains largely unaffected. This suggests that the single crystal primarily comprises of adjacent reentry folded structures rather than an intermediate model structure. Additionally, the Zhang group calculated that the fraction of the adjacent reentry folding for the solution‐grown single crystals is 91%‐95% by SMFS, which supports our results [39]. Therefore, we believe that most of the chains in single crystals formed at 35°C follow the adjacent reentry chain model.

The conclusion can also be confirmed by the SMFS results as shown in Figure S1, which provides folding information at the molecular level. The typical force‐extension curve shown in Figure 3c, when unfolding the folded chain, shows multiple peaks.  According to our previous research, the distance between adjacent peaks is approximately twice the thickness of a single crystal. Here, the average distance between each peak is about 26 ± 5 nm. The thickness of a PEO single crystal is about 10 nm, and considering that the PEO chain adopts a 7_2_‐helix conformation, the unfolded length of one folding cycle is about 13 nm, which is very close to the 26 ± 5 nm measured by SMFS. When the one‐fold of the single chain begins to be stretched, the force decreases to about 25 pN, which can be considered the force of the chain sliding in the loop region. Therefore, the stretching process of the single chain can be divided into several steps: (I) the cantilever attaches to the thiol end of the polymer chain, initiating the stretching, and the stretching force gradually increases from 0; (II) the cantilever begins stretching the first adjacent reentry folded partial chain, and the stretching force reaches its maximum; (III) the first adjacent reentry folded chain and loop undergo total stretching, causing a decrease in the stretching force, which means the adjacent intra‐chain interaction was broken [32]. The cantilever then moves on to stretch the second adjacent reentry folded chain, and the force increases again, repeating the process. The distance between points II and III in the curve is twice as thick as the crystal, corresponding to the stretch of a one‐fold partial chain containing two stems; (IV) this continues until the chain is fully stretched and assumes an extended configuration. Ultimately, the stretching length reached 124 nm, which is very close to the theoretical contour length of a single PEO (20 kg/mol) chain. This shows that in this crystalline structure, the stretching process of a single chain is independent and will not be affected by the surrounding chains, further supporting the conclusion that the single crystal adopts the adjacent reentry structure.

### 2.3 Chain‐Folding Analysis by SMFS for Low‐Temperature Crystallization

**FIGURE 4:**
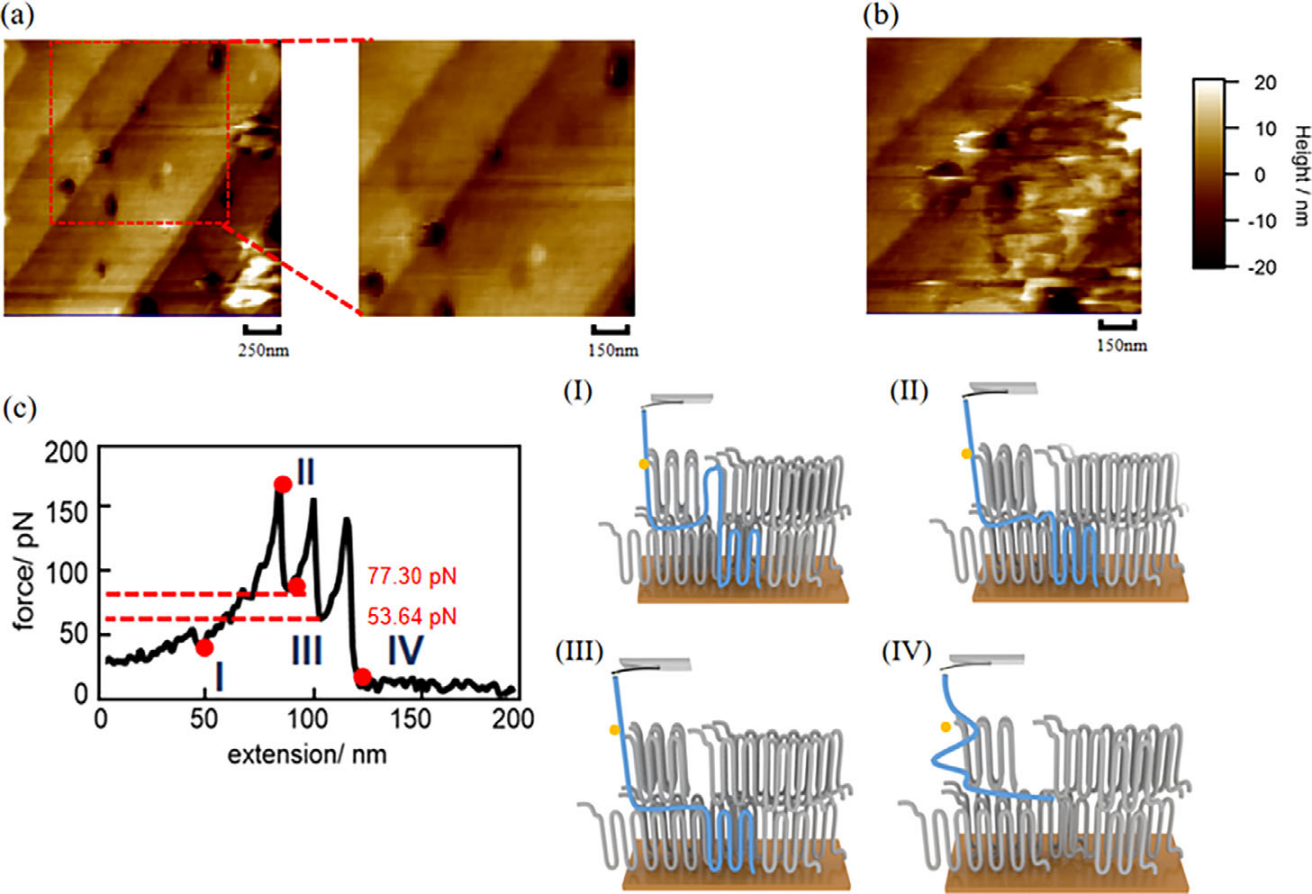
The morphology of the crystal formed at *T_c _= *10°C (a) before SMFS and (b) after SMFS in amyl acetate (liquid). (c) Force‐extension curve of a single chain in amyl acetate obtained in a crystal formed at *T_c _=* 10°C, and four steps of a single chain stretched by a gold‐coated cantilever in this sample.

However, the morphology of the crystal formed at *T_c _= *10°C before the SMFS experiment is distinctly different from that of the single‐layer crystal formed at *T_c _= *35°C. As shown in Figure 4a, the sample exhibits a multilayered stacked crystalline structure, resembling the morphology observed in Figure 2b. Similar to crystallization at 35°C, white granular structures are also on the surface. The formation of these white spots is likely due to the inhibition of chain‐end folding by terminal thiol groups. However, since the crystal consists of multiple stacked layers, most defects are buried within the structure, resulting in a lower density of white spots than the single‐layer crystal. Figure 4b illustrates the morphological changes in the sample after the SMFS experiment. Unlike the 35°C single‐layer crystal, the multilayer stacked structure was disrupted during stretching, leading to a significantly blurred morphology. If the chains in this crystal followed the adjacent reentry folding model, then the stretching of a single chain would leave only a tiny vacancy in its original position without affecting the surrounding structure (as observed in Figure 3b). However, the experimental results indicate a substantial difference between the interchain structure of this sample and the adjacent reentry model. Instead of folding in a 2D manner, we propose that the polymer chains in this sample adopt a more complex 3D interpenetrating configuration, where chains traverse multiple folded layers. As a result, the SMFS experiment disrupted the entire multilayer structure rather than leaving localized vacancies.

Further insights into the spatial configuration of the polymer chains are provided by the force‐extension curves in Figure S2, and Figure 4c shows one typical force‐extension curve. First, the force‐extension curve also shows three peaks and the distance of each peak is similar at 18 ± 2 nm. The thickness of a single layer of the crystal is 7 nm, as shown in Figure 2b. Similar to the previous discussion, considering that the PEO chain adopts a 7_2_‐helix conformation, the distance between adjacent peaks can be regarded as twice the length of the thickness of a single crystal. Thus, chain folding structures remain present even in the multilayered crystal formed at 10°C, chain folding structures remain present. However, compared to the adjacent reentry model, the stretching force required for chain extension is significantly higher, reaching 150 pN, which is considerably greater than the force observed for adjacent reentry folded structures in Figure 4c. In the 10°C crystallized sample, stretching a single chain requires overcoming not only interactions between adjacent folded chains but also additional interlayer interactions between chains in different crystalline layers. Furthermore, after the single chain is stretched to twice its original length, the force decreases to 53–77 pN, which remains notably higher than the 20 pN observed in the adjacent reentry folding model. This phenomenon can be attributed to restricted chain mobility, as the polymer chains experience constraints imposed by surrounding chains during interlayer sliding, leading to an increased force requirement. A typical stretching process is shown in Figure 4c, which can be divided into the following steps: (I) the cantilever attaches to the thiol end of the polymer chain, initiating the stretching; (II) since the front part of the chain is restricted by other folded chains, a larger force is required to pull out the first folded partial chain; then, as the folded chain is stretched, the force decreases, but in the indeterminate regions of the upper and lower layers of crystals, the tightening of the loop and the movement of the polymer segments still require a larger force, resulting in a force decrease to 70 pN, which is called chain traveling behavior between two layers; (III) as the first folded partial chain is stretched out of the crystal, the force increases again and begins to stretch the second folded partial chain. The distance between points II and III in the curve is approximately 18 nm, twice as thick as one layer, corresponding to the unfolding process; (IV) as the remainder of the chain is stretched, the other folded structures are pulled out together. Similarly, the stretching length is 126 nm, representing one intermediate structure stretched from the crystalline region. Based on the above experimental results, we propose that a multilayer‐interpenetrating intermediate structure is formed at a crystallization temperature of 10°C.

### 2.4 The Generation Mechanism of Multilayer‐Interpenetrating Intermediate Structure

**FIGURE 5:**
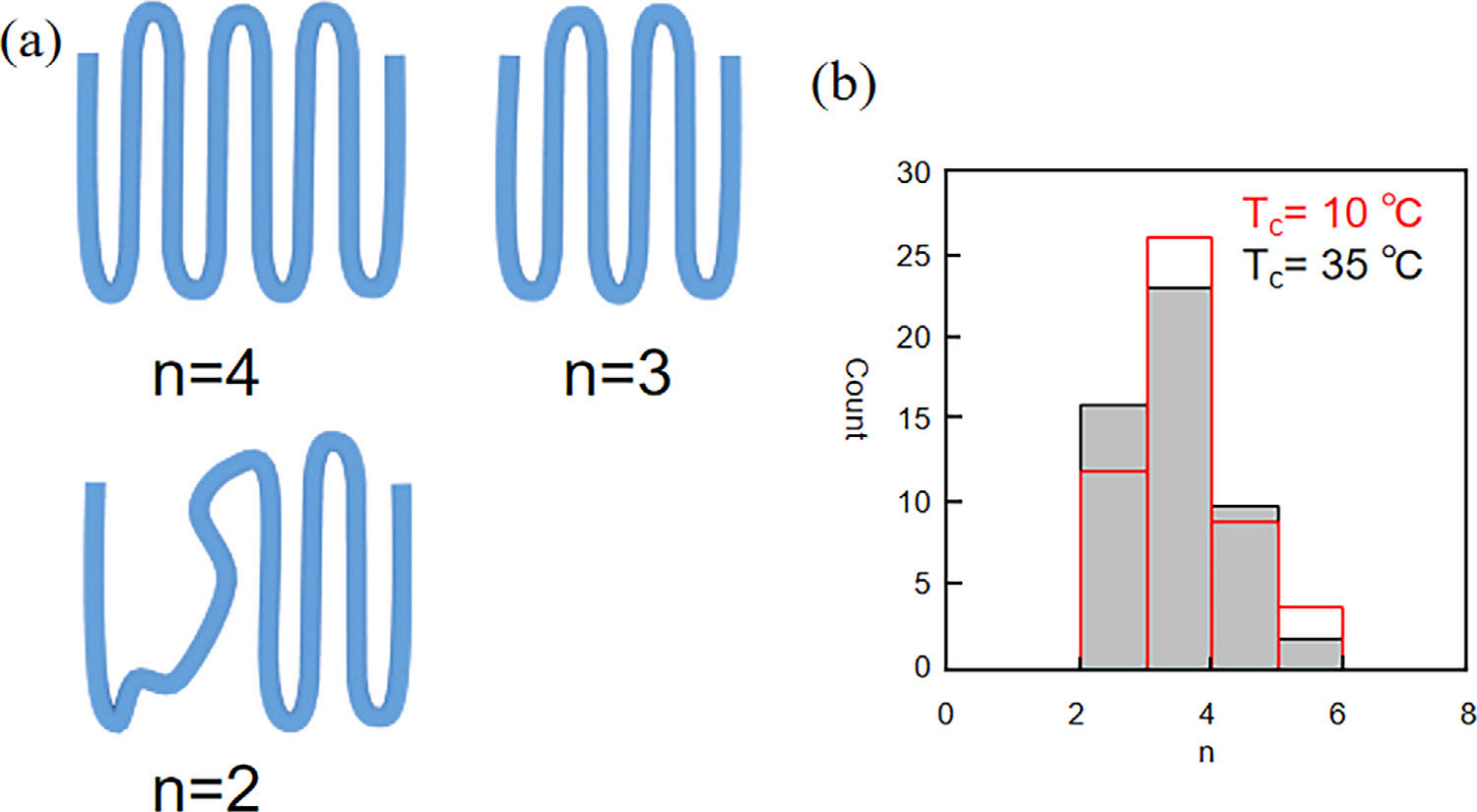
(a) The number of adjacent reentry folding events in three different chain structures. (b) Histogram of the number of peaks in the 50 single‐chain force‐extension curves from crystals formed at *T_c_* *= *10°C (red line and white region) and 35°C (black line and grey region) in amyl acetate (liquid).

Supercooling significantly affects the folding structure of the molecular chain. At a high supercooling state, the molecular chain tends to form intermediate models interspersed between multiple layers. In contrast, while at a low supercooling state, it mainly forms a single‐layered adjacent reentry model. To further explore the mechanism of the formation of the interlayered intermediate structure, we need to investigate the folding process of the molecular chain. As mentioned above, the force‐extension curves of SMFS can provide information on the folding of the polymer chain. We define <n> as the number of adjacent re‐entrant folding events. Figure 5a shows the different folding structures corresponding to <n> = 4, 3, and 2, respectively. The peak counts of 50 force‐extension curves obtained for crystals formed at *T_c_* *=* 10°C and 35°C are shown in Figure 5b. The results demonstrate that the value of n is almost constant regardless of the crystallization temperature. This, indicatinges that the number of adjacent reentrant folds in the nanocluster does not change with temperature, which contradicts the prediction of the traditional secondary nucleation theory (i.e., the degree of supercooling affects the number of folds in the molecular chain). However, considering the pre‐crystallization stage of the secondary nucleation theory, the initial folding process of the molecular chain is mainly dominated by the self‐folding mechanism. The polymer chains will self‐fold to form baby nuclei, which are not affected by kinetic factors. Therefore, we believe that at a large degree of supercooling, the molecular chain maintains its pre‐crystallization stage of folding throughout the crystallization process, regardless of the degree of supercooling.

**FIGURE 6:**
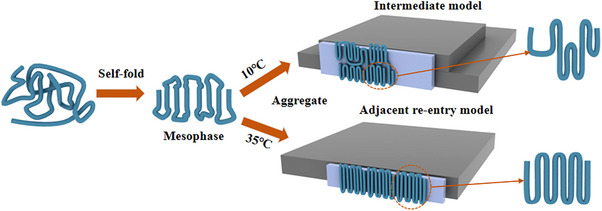
Schematic of two steps in the temperature‐dependent crystallization mechanism.

We refer to these early‐stage pre‐crystallization folded clusters as the “mesophase,” which is independent of *T_c_*. However, it is believed that polymer chains will form linear adjacent folding structures at relatively low supercooling while forming 3D folding structures to minimize surface free energy at large supercooling [2]. Therefore, the transitions from the mesophase to the final folded structure are primarily governed by the cluster aggregation process during the later stages of crystallization, as illustrated in Figure 6. At low *T_c_*, polymer chains undergo sliding and diffusion to minimize surface free energy, reducing the size of the final folded chain structure. During this process, chains may interpenetrate multiple clusters, ultimately forming multilayer crystalline structures. Conversely, at high *T_c_*, larger final folded chain structures can form, allowing the folded chain length within the mesophase clusters to increase during aggregation, forming a thicker single‐layer crystalline structure. To further investigate the impact of crystallization temperature on the formation of the final folded chain structure, we analyzed the effect of the nucleation barrier based on classical nucleation theory.

In line with Muthukumar's analysis, we also analyzed the nucleation barrier [22]. According to classical nucleation theory, the nucleation barrier arises from the competition between bulk and surface energies.

$$\Delta F=R^{2}\;\Delta \mu -4R\sigma$$

whereΔF is the free energy of forming a nucleus of size R, with size, *R* with Δμ and σ being the bulk and lateral surface energies, respectively. By assuming $\frac{\partial F}{\partial R}$ = 0 and bulk energy can be linearized with respect to supercooling ΔT when approaching *T_m_* [40], the critical nucleus size $R_{C}$ at a fixed concentration is obtained as follows:

$$R_{C}=\frac{2\sigma T_{m}}{E\Delta T}$$

where *E* represents the unit of edge binding energy. Therefore, increasing *T_c_* will lead to smaller supercooling ΔT and larger $R_{C}$.

Applying this formula to single chains, polymer chains adopt the adjacent reentry model of large *Rc* at high *T_c_*, while forming the intermediate model of low *Rc* at low *T_c_*. In addition, it was found that the fraction of adjacent reentry folding will decrease as *T_c_* decreases by DQ‐NMR [41]. Thus, the different final chain structures can be attributed to the temperature‐dependent chain behavior. At high *T_c_*, folded chains in the mesophase will become regular and thicker, resulting in a thicker monolayer with a high fraction of adjacent reentry folding. At low *T_c_*, the folded chains will slide and intersperse with other folded chains, and although the folding number <n> remains the same, the thickness of a layer decreases. Finally, it forms an intermediate model with a low fraction of adjacent reentry folding.

## 3 Conclusion

We performed SMFS experiments based on AFM in the mode of FV, realizing the simultaneous acquisition of the microstructure of a single polymer chain and structural information in single crystals. By comparing the changes in single‐crystal morphology before and after SMFS, we were able to locate a specific single polymer chain and further obtain its spatial conformation inside the crystal. This conformational information can be used to infer the specific form of the chain folding structure and its formation mechanism in different crystal types. Based on this method, we have deeply investigated the structural changes of PEO single chains at different crystallization temperatures. The experimental results show that the morphology of single PEO crystals formed in dilute solution results from the temperature‐dependent chain structures.

In the early stage of crystallization, polymer chains self‐fold to form many small clusters as a mesophase. The number of adjacent reentry folding events‐reflecting how chains fold back upon themselves inside the cluster‐remains consistent at different *T_c_*, indicating that the self‐folding process is independent of *T_c_*. However, *T_c_* does play a role in the subsequent aggregation of these clusters and of the chain structure inside. At a lower *T_c_* of 10°C, the self‐folded chain structure becomes interspersed with a 3D intermediate structure on both the upper and lower crystal surfaces. In contrast, at 35°C, the chains form a more uniform adjacent reentry structure, leading to a thicker single crystal. These temperature‐dependent chain structures highlight how thermal conditions affect chain behavior during crystallization. In summary, this method successfully obtained the 3D conformational information of single chains in crystallization, providing important insights for a deep understanding of early crystallization behavior at the molecular level, and providing an important experimental reference for further improving the kinetic theory of crystallization polymers.

## 4 Experimental Section

### 4.1 Materials

Amyl acetate was purchased from Fujifilm, JP. Ultra‐pure water was used to prepare all aqueous solutions. Polyethylene oxide (PEO) with double thiol ends (HS‐PEO‐SH), M_n _= 20,000 g/mol, was purchased from NOF Corporation, JP.

### 4.2 Sample Preparation

Overall, 5 mg of PEO powder was dissolved in 10 g of amyl acetate (0.05 wt.%) at 68°C. Once fully dissolved, the solution was transferred to an oil bath at 35°C for 1 min to maintain a consistent cooling rate. Subsequently, a portion of the PEO solution was maintained at 35°C, while another portion of the solution was rapidly cooled to 10°C and left to stand for 10 h to complete the isothermal crystallization process.

The SMFS samples were prepared as follows: the different temperature crystallization solutions were deposited on the surface of the gold‐plated substrate and left to stand for 5 min. The remaining solution was then thoroughly rinsed with pure amyl acetate multiple times. The samples were dried in a desiccator for 2 h before being removed for experiments. All Au‐coated substrates were placed into a UV ozone cleaner (UV253, Fligen, JP) for at least 20 h prior to use.

### 4.3 SMFS

All AFM‐based SMFS experiments in air and liquid were performed using an AFM Multimode 8 (Bruker, US). Silicon cantilevers (BL‐AC40TS‐C2, 0.09 N/m, OLYMPUS, Japan) were first gold‐plated by the MC1000 Ion Sputter Coater (HITACHI, Japan). The thin Au coating was deposited on the cantilever to make it electrically conductive and avoid charge build‐up during observation in AFM. Then, the Au‐coated cantilever was employed to obtain sample topographies and conduct SMFS experiments in liquid at room temperature. Initially, AFM images of PEO single crystals were acquired using tapping mode, enabling the analysis of crystal morphology and the identification of specific areas. After pinpointing a crystal region, the system was switched to force‐volume mode, allowing for the random extraction of molecular chains within the chosen crystal region through multiple scans. Force‐extension curves were collected at a single pulling speed of 10 µm/s to ensure comparability during the stretching process. Following this, the system was switched back to tapping mode to capture morphological images of the same region after SMFS. By comparing these images with those taken prior to SMFS, we determined where a single polymer chain had been successfully extracted. By calibrating the spring constant and deflection sensitivity of the cantilever, the tensile force‐extension curve of a stretched single‐molecule chain is obtained. The cantilever's deflection sensitivity was calibrated on a sapphire substrate, and the spring constant was corrected using thermal tuning analysis.

## Conflicts of Interest

The authors declare no conflicts of interest.

## Supporting information




**Supporting File**: marc70212‐sup‐0001‐SuppMat.docx.

## Data Availability

The data that support the findings of this study are available from the corresponding author upon reasonable request.
