# Supplementary material for: Temperature‐Dependent Chain Structures during Solution‐Grown Crystallization via Atomic Force Microscopy
Source: Macromol Rapid Commun. 2026 Jan 26;47(7):e00842. doi: 10.1002/marc.202500842 (PMC13047474; doi:10.1002/marc.202500842)
Supplement: Supplementary file 1 — Supporting File: marc70212‐sup‐0001‐SuppMat.docx. [file MARC-47-e00842-s001.docx]

**Temperature-Dependent Chain Structures during Solution-Grown Crystallization via Atomic Force Microscopy**

Dingrui Wang, Xiaobin Liang, Ken Nakajima*

*Department of Chemical Science and Engineering, School of Materials and Chemical Technology, Institute of Science Tokyo, Ookayama 2-12-1, Meguro-ku, Tokyo 152-8550, Japan*

**Support information:**

**Force-extension curves of single chain obtained in crystal**

We conduct SMFS in FV mode and obtain enough force-extension curves to infer the structure. And we choose one specific force-extension curve as shown in Figure 3(c) and 4(c) to analyze the SMFS process. To ensure reproducibility, we present other force-extension curves obtained on crystal formed at T_c_= 35 ℃ (Figure S1) and T_c_= 10 ℃ (Figure S2)


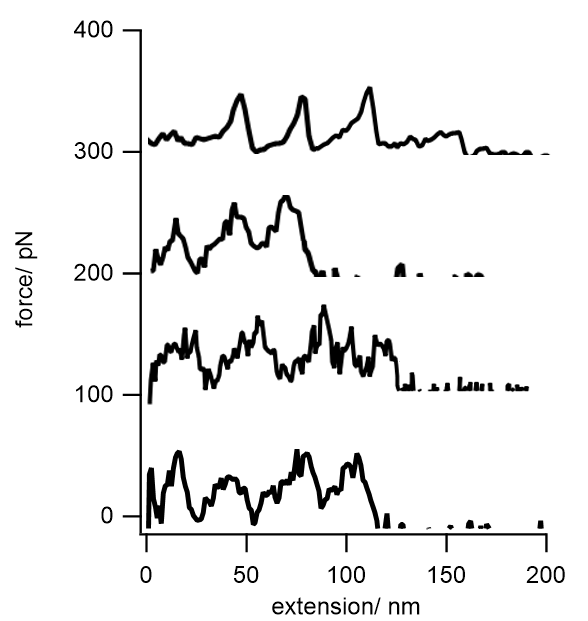


**Figure.S1** Force-extension curves obtained on crystal formed at T_c_= 35 ℃


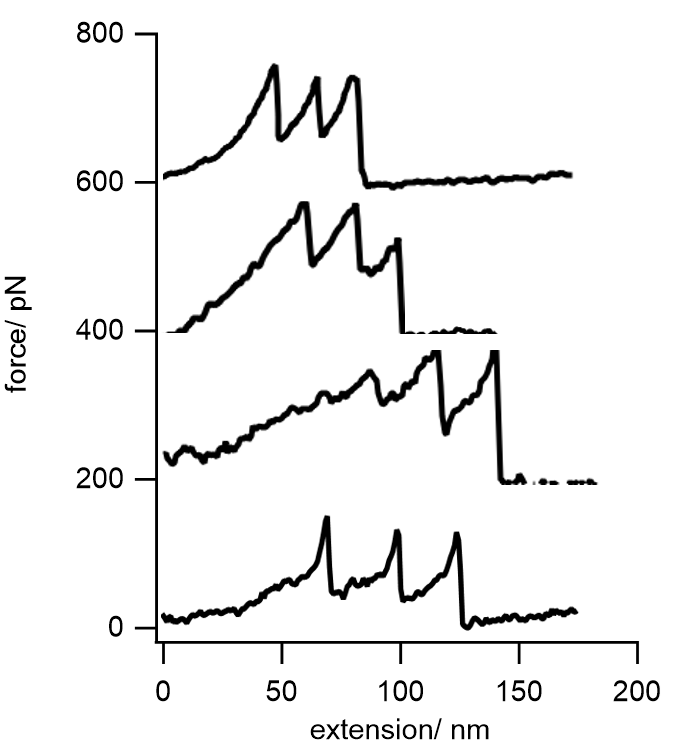


**Figure.S2** Force-extension curves obtained on crystal formed at T_c_= 10 ℃
